# The chemical stoichiometry characteristics of plant-soil carbon and nitrogen in subtropical *Pinus massoniana* natural forests

**DOI:** 10.1038/s41598-024-55740-z

**Published:** 2024-02-29

**Authors:** Yunxi Xiang, Ping Pan, Xunzhi Ouyang, Hao Zang, Jinfeng Rao

**Affiliations:** 1 Chongqing Three Gorges University, Wanzhou, 404100 China; 2 Key Laboratory of National Forestry and Grassland Administration for the Protection and Restoration of Forest Ecosystem in Poyang Lake Basin, Jiangxi Agricultural University, Nanchang, 330045 China; 3 College of Forestry, Jiangxi Agricultural University, Nanchang, 330045 China

**Keywords:** Plant sciences, Ecology

## Abstract

Ecological stoichiometry is essential for understanding changes in ecosystem structure and nutrient cycling in forest ecosystems. However, the stoichiometric characteristics of carbon (C) and nitrogen (N) in different organs or layers, such as leaves, branches, trunks, roots, understory vegetation, litter, and soil within a forest ecosystem, have remained poorly understood. In this study, four age groups of *Pinus massoniana* natural forest including young, middle-aged, near-mature, and mature were selected as research subjects to illustrate the C and N stoichiometry interactions among different layers and organs in the forest ecosystem. The results showed that the average C and N concentrations in the leaves of the tree layer, shrub layer, and herb aboveground parts (HAP) were higher than that of other tree and shrub organs, as well as the herb underground parts (HUP), respectively. The N concentrations of tree branches and trunks showed a trend of increase first and decrease later from young to mature phases, but the C:N ratios presented an opposite trend. The C concentrations.in all tissues in shrubs showed a first decline and then a rise with age. As age progressed, the N concentration in each ecosystem layer increased gradually and demonstrated high synergy. The mineralization of organic matter in the soil was generally slow. The C concentrations in the understory vegetation layer were significantly positively correlated with the C concentrations in the litter layer but negatively correlated with the soil layer, and the C concentrations in the litter layer were also significantly negatively correlated with the C concentrations in the soil layer. The research findings can provide a reference basis for the formulation of nutrient regulation and sustainable management measures in the natural forests of *P. massoniana* in the study area.

## Introduction

The world’s forests provide countless ecosystem services essential to humans’ continued existence and well-being^1^. With rapid socio-economic development and a growing population, the demand for forest resources has increased, increasing pressure on forest ecosystems^2,3^. The energy flow and material cycling of the entire forest ecosystem are largely influenced by vegetation and soil. Sustainable land utilization is promoted by the interaction between soil and vegetation, which stabilizes the soil and increases nutrients^4^. Litter decomposition is a crucial process in the forest ecosystem due to its close association with ecological processes such as soil formation, and nutrient cycling^5^. The continuous decomposition of litter constantly replenishes the soil with mineral nutrients, so that the limited nutrients are continuously recycled by plants, which not only maintains soil fertility but also maintains the balance of the forest ecosystem^6^. The cycling of nutrients plays a fundamental role in strengthening the structure and function of forest ecosystem^4^. Carbon (C) and nitrogen (N) affect biogeochemical cycling and critical ecological processes, and play a vital role in the formation and maintenance of subattributes^7,8^. The ecological stoichiometry theory seeks to investigate the balance of various nutrients (especially C and N) in ecosystems, and has been widely applied in terrestrial ecosystems^9,10^. Numerous studies have examined C and N dynamics during stand development^11^, and thus, present the possibility of quantifying the temporal patterns of C:N stoichiometry over the age sequence of different ecosystems. In northeastern China’s natural temperate forest, for instance, it has been shown that the ratios of C:N in leaves and roots decrease with the stand age. The C:N ratios in branches and fine roots underwent significant changes during forest development^12,13^.

At present, studies on the C and N stoichiometry of forest ecosystems are mainly focused on different forest types or the C and N nutrient concentrations of individual layers or plant tissues. For example, the study of the soil C, N stoichiometry in a mature subtropical broadleaf forest found that the C:N ratios decreased with the increasing soil depth^14^, and it summarized the reports of the soil C, N stoichiometry in tropical forests on Hainan Island, China^15^. Liu et al^11^ reported that the soil organic C and total N concentrations increased significantly with afforestation age and peaked in the 40–50-year-old stand in *Robinia pseudoacacia* forests on the Loess Plateau, China. They also demonstrated that the age of the forest stand had different influences on the concentrations of C and N in leaves, leaf litter, and fine roots. The most obvious trend was that the concentration of N in leaves and litter increased with age. Chang et al.^16^ studied the patterns and driving factors of leaf C and N stoichiometry in two forest types with different stand ages in a mid-subtropical zone and found that latitude and mean annual precipitation were the main controlling factors affecting the variations in leaf C and N status in natural evergreen broadleaved forests. Shi et al.^17^ noted that the foliar and root C:N ratios were significantly higher in overstory trees than in understory shrubs and herbs. The C:N ratios of leaves and rhizosphere soils across all species were greatly influenced by forest type. The C:N ratio in the leaves was positively correlated with the rhizosphere soil, while the ratio in the roots was negatively correlated with the rhizosphere soil. Besides, the studies are few about the C and N stoichiometric characteristics of different successional stages in the whole forest ecosystem and the correlation between the C and N cycling processes of different layers^18,19^.

*Pinus massoniana* Lamb. is a typical native coniferous tree species in subtropical China, and is considered to be one of the most innovative tree species for vegetation restoration. It has strong adaptability and tolerance to drought and poor soil, and it has a wide distribution area. *P. massoniana* forest is a significant forest type in Jiangxi Province and is crucial in regional nutrient cycling and coping with climate change^20^.

In this study, we addressed the following questions: (1) What is the distribution and characteristics of C and N stoichiometry in the tree layer, understorey vegetation layer, litter layer, and soil layer of natural *P. massoniana* at different stand ages? (2) Are there any correlations between the stoichiometry of C and N in different ecosystem layers?

## Materials and methods

### Study area

The study area was located in Ganzhou City, Southern Jiangxi Province, China (24° 29′–27° 09′ N, 113° 54′–116°38′ E). The total land area is 166,900 km^2^. The climate in this region is a humid subtropical monsoonal climate with four distinct seasons, and a distinct wet season from April to June. The average temperature throughout the year is between 19.1 and 20.8 °C. The average amount of precipitation per year is around 1580 mm. The frost-free period lasts for 240–307 days. The average number of sunshine hours per year is 1636.3 h. Mountains and hills are the dominant features of the landscape. The soils are mostly red soil and red-yellow soil according to the Chinese soil classification, which are classified as ferralsols in the World Reference Base for Soil Resources. The main parent rocks are granite, gneiss, phyllite, and slate. The forest coverage rate in the area is 76.2%, due to its rich forest resources. The natural *P. massoniana* forest area reaches 928,000 ha, accounting for 36.0% of the city's forest land area^21^.

### Description of sample plots

The forest was classified in four distinct growth stages (stand ages) on the basis of the growth characteristics of the natural *P. massoniana* forest: young forest (≤ 20 years), middle-aged forest (21–30 years), near-mature forest (31–40 years), and mature forest (41–60 years). To ensure that all plots were comparable, they all sloped with a middle slope, had a sunny slope, had granite as their pedogenic process, had a soil thickness of 70–100 cm, and had ferralsol as their soil type. There were five random replications created for every age group, totaling 20 plots. Each plot measured 0.08 ha (28.28 m × 28.28 m, Table 1). The cone method was used to measure the age of three trees that represented the age of the sample plot, and the average age of the three trees was used to represent the age of the sample plot. For each plot, the DBH (diameter at breast height (1.3 m), minimum ≥ 5 cm; lower values were classified as understory vegetation) of each tree was measured individually, and stand and site factors were determined. The plots were divided into 2 m × 2 m subplots for shrubs, while three 1 m × 1 m subplots were set up along a diagonal line for litter inside the plots. The investigation focused on the type, quantity, and coverage factors of undergrowth vegetation. A soil profile with a depth of 100 cm was dug in each plot, and the soil samples were taken separately from the depths of 0–10, 10–20, 20–30, 30–50, and 50–100 cm. The samples of each soil layer for soil bulk density were collected using a cutting ring (volume of 100 cm^3^). At each depth, approximately 1 kg of soil was returned to the laboratory for natural air drying, and it was used to determine the soil nutrient concentrations.

**Table 1:** Basic characteristics of different age groups (means ± standard errors).

Age group	Average age (a)	Average DBH (cm)	Average height (m)	Average stand density (N ha^−1^)	Canopy density	Shrub coverage (%)	Herb coverage (%)	Soil pH
Young forest	16.2 ± 1.24	8.3 ± 0.45	6.4 ± 0.18	1651.3 ± 133.51	0.5 ± 0.06	4.8 ± 0.27	81.3 ± 3.40	4.79 ± 0.14
Middle-aged forest	26.0 ± 0.95	11.3 ± 0.37	8.9 ± 0.32	1112.5 ± 53.33	0.6 ± 0.05	9.8 ± 0.23	57.7 ± 4.82	4.92 ± 0.10
Near-mature forest	34.2 ± 1.24	15.7 ± 0.55	13.5 ± 0.16	886.2 ± 52.66	0.6 ± 0.03	38.6 ± 5.58	60.0 ± 4.36	5.01 ± 0.23
Mature forest	46.0 ± 1.87	20.8 ± 0.69	15.7 ± 0.25	740.0 ± 46.67	0.7 ± 0.06	10.9 ± 1.47	68.0 ± 4.37	5.00 ± 0.15

### Sample collection and processing

Standard representative trees that had a DBH and height that matched or was similar to the average DBH and height of the sample plots were selected based on measured DBH data. The selected trees were examined for tissue samples, which included leaves, branches, stems, and roots. Leaf and branch samples were taken from the middle of the crown. Stem samples were taken from the middle of the trunk. Root samples were manually excavated at approximately 1 m depth within a radius of 1 m from the tree center. The biomass of understory plants, consisting of herbs, shrubs and trees with a DBH of less than 5 cm, was measured in each sample plot using destructive sampling techniques. The fresh weight of shrub biomass tissues, which comprise leaves, branches, and roots, and herb biomass components, which comprise aboveground and underground parts, was separately collected and weighed to determine their fresh weight. The fresh weight of the litter was determined by collecting and weighing it. Approximately 200 g of each sample was then taken back to the laboratory and dried in an oven at 65 °C for moisture concentration and nutrient concentration analyses. A modified Walkley–Black-Acid–Dichromate FeSO_4_-Titration Method^12,22^ was utilized to assess the organic C content of soil and plant and litter samples. The total N concentration of the soil, plant, and litter samples was determined using the modified Kjeldahl method^23^.

### Data analysis and processing

The C and N concentrations of the tree layer, understory vegetation layer, and litter layer in the sample plot were calculated by the average weight according to the corresponding concentrations of C and N in each part^24^. The calculation formula is as follows:

1 $$W_{i} = \frac{{\sum\nolimits_{j = 1}^{n} {C_{{{\text{ij}}}} \times BD_{{{\text{i}}j}} } }}{{\sum\nolimits_{{{\text{j = }}1}}^{n} {BD_{{{\text{ij}}}} } }}$$

where * W*_*i*_ represents the C and N concentrations (g kg^−1^) of layer *i* (*i* corresponds to the tree layer, understory vegetation layer and litter layer), and *C*_*ij*_ and *BD*_*ij*_ represent the C and N concentrations (g kg^−1^) and biomass density (Mg ha^−1^) of tissues *j* in layer *i*, respectively. When* i* is the tree layer, n = 4, *j* = leaf, branch, stern, root; when* i* is the understory vegetation layer, *n* = 5, *j* = shrub leaf (BL), shrub branch (BB); shrub root (BR); herb aboveground part (HAP); and herb underground part (HUP). The biomass density is determined by the amount of biomass per unit area. Allometric equations were used to calculate the biomass of each tree tissue^25^. The biomass of the understory vegetation and litter was calculated on the basis of the fresh weight and moisture concentration.

The C and N concentrations in the soil layer (0–100 cm) are calculated as follows:

2 $$T{ = }\frac{{\sum\nolimits_{{{\text{i = }}1}}^{5} {C_{i} \times B_{i} \times D_{i} } }}{{\sum\nolimits_{{{\text{i = }}1}}^{5} {B_{i} \times D_{i} } }}$$

where *T* represents the concentration (g kg^−1^) of C and N in the soil layer (0–100 cm), and* C*_*i,*_* B*_*i*_ and *D*_*i*_ represent the concentration (g kg^−1^) of C and N, bulk density (g cm^-3^), and depth (cm) in the soil layer, respectively.

All the stoichiometric ratios were calculated on a mass basis. All statistical analyses were performed using a SPSS 19.0 (SPSS Inc, Chicago, IL, USA) software. The differences in C and N concentrations and their stoichiometric ratios in different stand ages, at different layers, and in different tissues or organs were analysed using one-way analysis of variance (ANOVA). Correlation heat map analysis was used to analyse the correlations between C and N concentrations and their stoichiometry in all layers.

## Results

### The concentrations of C and N and the distribution patterns of C:N ratio among different tissues of vegetation

**Figure 1:**
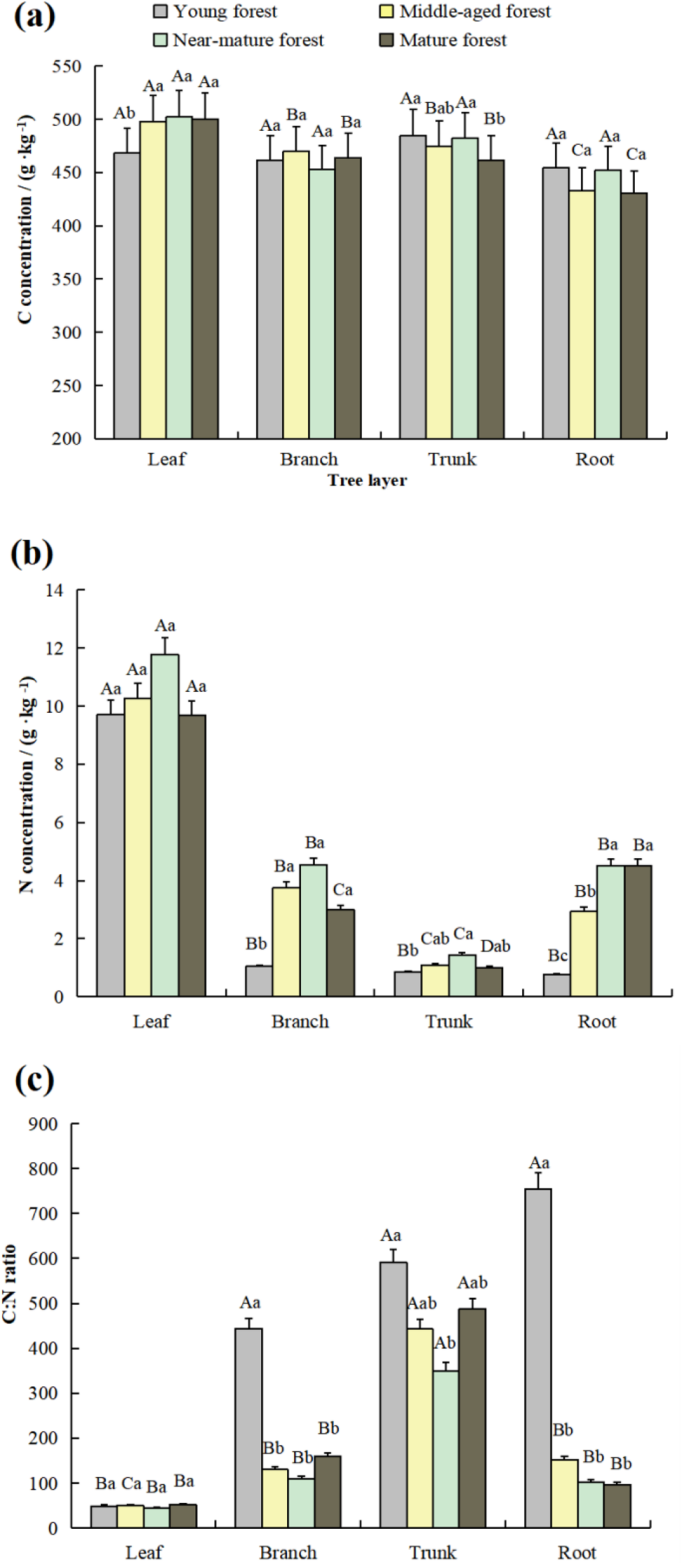
The C (**a**) and N (**b**) concentrations and C:N ratios (**c**) of the tree layer. Significant differences among organs can be identified by using different capital letters at *p* < 0.05. At *p* < 0.05, there are significant differences in lowercase letters among stand-alone ages.

Different vegetation layers, stand ages, and organs (plant tissues) had significantly affected the C and N concentration and C:N ratio distribution. As illustrated in Table 2, in the tree layer, the table showed that the average C concentration in the leaf was the highest (492.01 g kg^−1^), and that in the root was the lowest (442.54 g kg^−1^). The C concentrations of leaves in the middle-aged and mature forests were significantly higher than those of roots (Fig. 1). The average N concentration in the leaf was the highest (10.36 g kg^−1^), while the average C:N ratio in the leaf was the lowest (48.27). The N concentrations in all plant tissues showed a progression of increasing and then decreasing with the age. The differences in N concentrations in leaves among these stand ages were not significant, while remarkable changes in branch and root appeared in the young forest, in which the N concentrations were significantly lower than those in middle-aged, near-mature, and mature forests (*p* < 0.05). The N concentrations in the leaves of each stand age were significantly higher than those in branches, trunks and roots (*p* < 0.05). Stand ages did not have any effect on the C:N ratios in leaves in the study. The C:N ratios of branchs and roots in the young forest were significantly higher than those in the other stand ages (*p* < 0.05). The leaf C:N ratios in each stand age were significantly lower than those in the trunk (*p* < 0.05).

**Table 2:** Vegetation layer organs, mean C and N concentrations, and C:N ratios of four stand ages.

Variable	Organ	C (g kg^−1^)	N (g kg^−1^)	C:N
Tree layer	Leaf	492.00	10.36	48.27
	Branch	462.01	3.08	210.55
	Trunk	475.60	1.10	467.83
	Root	442.54	3.18	276.16
Understory vegetation layer	BL	401.96	14.33	28.91
	BB	396.12	6.29	65.06
	BR	367.82	6.69	59.06
	HAP	464.47	11.73	40.92
	HUP	339.91	6.22	57.23

**Figure 2:**
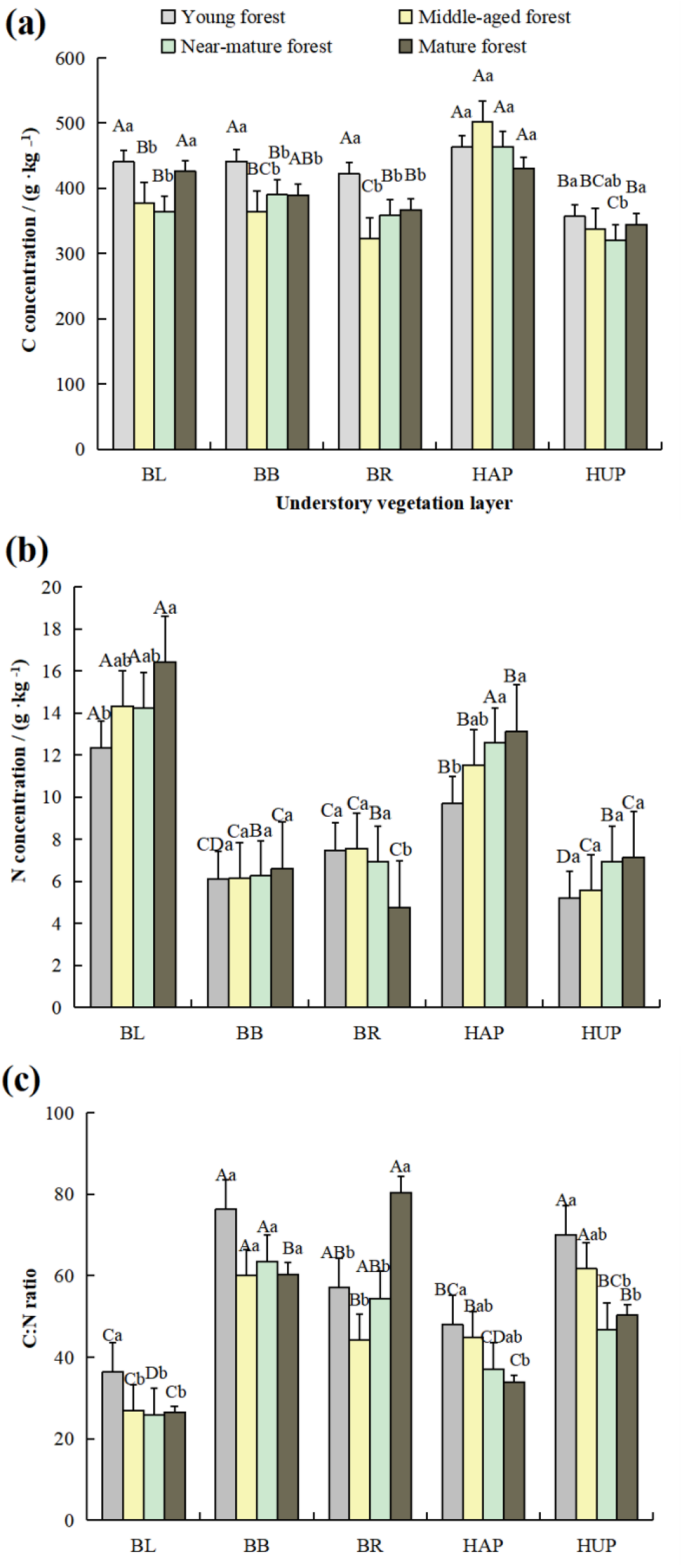
The C (**a**) and N (**b**) concentrations and C:N ratios (**c**) of the understory vegetation layer. Different capital letters represent significant differences among organs at *p* < 0.05. Different lowercase letters represent significant differences among stand-alone ages at *p* < 0.05. BL, shrub leaf. BB, shrub branch. BR, shrub root. HAP, herb aboveground part. HUP, herb underground part.

In the understory vegetation layer, as illustrated in Table 2, the C:N ratios of BR and HUP of 59.06 and 57.23, respectively, were higher than those of the average concentrations of BL and HAP of 28.91 and 40.92, respectively. The C concentrations in BL, BB, BR, and HUP all showed a trend of declining then increasing with the stand age, and the C concentrations for the near-mature forest in BL, BB, BR and HUP were significantly lower compared with those of the young forest (*p* < 0.05, Fig. 2). At each age, the C concentrations of HAP were significantly higher than those of HUP. In all age groups, there was a significant difference in the N concentrations of BL and HAP compared to those of BR, BB, and HUP (*p* < 0.05). There was a slight decrease in the C:N ratios of BB with increased stand age, but it was not significant.

### Soil C, N and C:N ratio distribution pattern in four stand ages vertically

**Figure 3:**
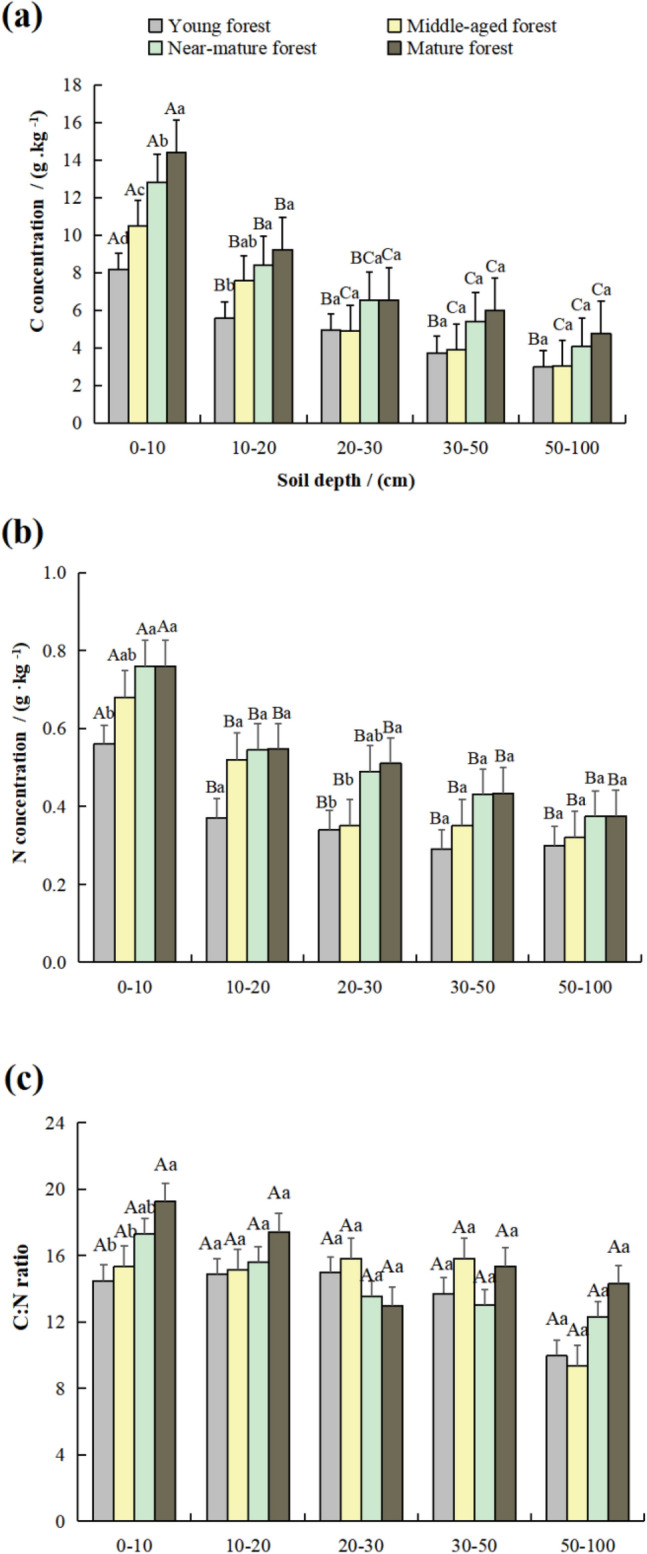
The C (**a**) and N (**b**) concentrations and C:N ratios (**c**) at different soil depths. Different capital letters represent significant differences among soil layers at *p* < 0.05. Different lowercase letters represent significant differences among stand-alone ages at *p* < 0.05.

The concentrations of soil carbon (C) and nitrogen (N), as well as the C:N ratios, were significantly influenced by stand age at different soil depths (Fig. 3). Specifically, C and N concentrations of the 0–10 cm soil depth constantly rose with stand age, which finally reached the top at 14.41 and 0.76 g kg^−1^, respectively, which were the highest and significantly higher than those of the other soil layers (*p* < 0.05). The C and N concentrations in 10–20, 20–30, 30–50, and 50–100 cm showed similar trends with the age. However, the C and N concentrations gradually decreased with increased soil depth, and there was no significant difference between the 20–30 and 50–100 cm soil depths. The C:N ratios of the 0–10 cm soil depth constantly rose with stand age, which finally reached the top at 19.24, and the C:N ratios in mature forest were significantly higher than those in young forest. Although the C:N ratio fluctuation was observed at 10–20, 20–30, 30–40, and 40–50 cm soil depths, the change was much less significant with increased stand age. These soil depths did not have any significant differences in their C:N ratios.

### C and N concentrations and C:N ratio distribution patterns among various layers of the ecosystem

Different layers had significantly affected the C and N concentrations and C:N ratios. As illustrated in Table 3, the C concentrations of the tree layer showed an increase and subsequently a decrease with stand age, which reached the highest level in near-mature forests. On the other hand, the C:N ratios of the tree layer presented an opposite trend. The C concentrations and C:N ratios of the tree layer were the highest of all stand ages and were also significantly higher than those of the other layer (*p* < 0.05). Although a constant rise took place in the C and N concentrations and C:N ratios of the soil layer with stand age, the figures for those remained the minimum among ecosystem components and were significantly lower than those of the other layer (*p* < 0.05). The N concentrations showed a trend of rising in all ecosystem components with stand age, which all reached the top at mature forest. There was a significant difference in N concentrations between different components of the ecosystem: litter layer > understory vegetation layer > tree layer > soil layer (*p* < 0.05). The C concentrations and C:N ratios of the understory vegetation and litter layers showed a trend of decline with stand age, but there was no significant difference.

**Table 3:** Ecosystem C and N concentrations and C:N ratios.

Variable	Age group	Tree layer	Understory vegetation layer	Litter layer	Soil layer
C (g kg^−1^)	Young forest	461.71	417.79	420.42	3.81
	Middle-aged forest	469.71	401.32	413.86	4.46
	Near-mature forest	475.92	388.21	405.33	5.39
	Mature forest	470.79	385.69	397.41	6.23
	Mean	469.53 a	398.25 b	409.26 b	4.98 c
N (g kg^−1^)	Young forest	2.17	7.51	9.67	0.32
	Middle-aged forest	2.38	8.84	10.51	0.37
	Near-mature forest	2.75	9.27	10.98	0.43
	Mature forest	3.38	9.48	11.01	0.44
	Mean	2.67 c	8.78 b	10.54 a	0.39 d
C:N	Young forest	551.79	60.30	43.60	11.92
	Middle-aged forest	328.07	48.67	39.59	12.38
	Near-mature forest	274.25	46.39	36.93	13.09
	Mature forest	352.26	45.73	36.20	15.00
	Mean	376.59 a	50.28 b	39.08 b	13.10 c

Different lowercase letters represent significant differences among ecosystem organs (*p* < 0.05).

### The correlation of various layers of the ecosystem

**Figure 4:**
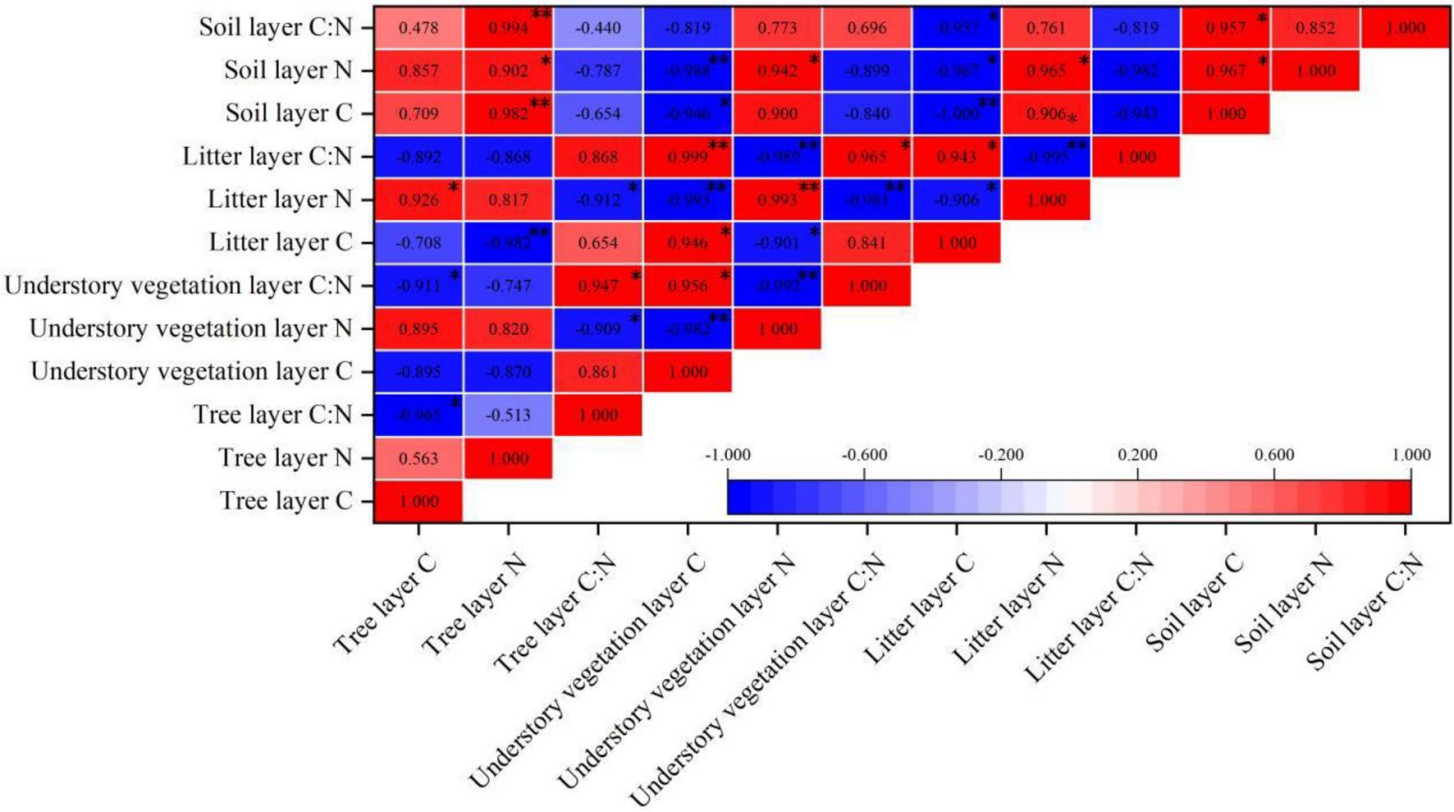
The C and N concentrations and C:N ratio correlations within each layer of the ecosystem. * represents a significant correlation at *p* < 0.05; ** represents a significant correlation at *p* < 0.01.

The C and N concentrations and the C:N ratio showed significant correlations at various layers of the ecosystem as displayed in Fig. 4. The Pearson correlations indicated that soil layer N was significantly correlated with the tree layer of N (r = 0.902, *p* < 0.05), understory vegetation layer of C (r = − 0.988, *p* < 0.01), understory vegetation layer of N (r = 0.942, *p* < 0.05), litter layer of C (r = − 0.967, *p* < 0.05), litter layer of N (r = 0.965, *p* < 0.05), litter layer of C:N (r = − 0.982, *p* < 0.05), and soil layer of C (r = 0.967, *p* < 0.05). Litter layer of N was significantly correlated with tree layer of C (r = 0.926, *p* < 0.05), tree layer of C:N (r = − 0.912, *p* < 0.05), understory vegetation layer of C (r = − 0.993, *p* < 0.01), understory vegetation layer of N (r = 0.993, *p* < 0.01), understory vegetation layer of C:N (r = − 0.981, *p* < 0.01), and litter layer of C (r = − 0.906, *p* < 0.05). Litter layer of C:N was significantly correlated with the understory vegetation layer of C (r = 0.999, *p* < 0.01), understory vegetation layer of C (r = − 0.989, *p* < 0.01), understory vegetation layer of C:N (r = 0.965, *p* < 0.01), litter layer C (r = 0.943, *p* < 0.05), and litter layer of N (r = − 0.995, *p* < 0.01). Soil layer of C was significantly correlated with the tree layer of N (r = 0.982, *p* < 0.01), understory vegetation layer of C (r = − 0.946, *p* < 0.05), litter layer of C (r = − 1.000, *p* < 0.01), litter layer of N (r = 0.906, *p* < 0.05), litter layer of C:N (r = − 0.943, *p* < 0.05). Understory vegetation layer of C:N was significantly correlated with the tree layer of C (r = − 0.911, *p* < 0.05), tree layer of C:N (r = 0.947, *p* < 0.05), understory vegetation layer of C (r = 0.956, *p* < 0.05), and understory vegetation layer of N (r = − 0.992, *p* < 0.01). Litter layer of C was significantly correlated with the tree layer of N (r = − 0.982, *p* < 0.01), understory vegetation layer C (r = 0.946, *p* < 0.05), and understory vegetation layer of N (r = − 0.901, *p* < 0.05). Soil layer of C:N was significantly correlated with the tree layer of N (r = 0.994, *p* < 0.01), litter layer of C (r = − 0.957, *p* < 0.05), and soil layer of N (r = 0.957, *p* < 0.05). Understory vegetation layer of N was significantly correlated with the tree layer of C:N (r = − 0.909, *p* < 0.05), and understory vegetation layer of C (r = − 0.982, *p* < 0.01). Tree layer of C:N was significantly correlated with the tree layer of C (r = − 0.965, *p* < 0.05).

## Discussion

### C and N concentrations and C:N ratio distribution patterns among various organs and layers of the ecosystem

In this study, the C and N concentrations and C:N ratio distribution patterns among various organs and layers of the ecosystem were observed. The average C concentrations of the tree layer leaves, shrub layer leaves, and HAP were higher than those of other tree organs, shrub organs, and HUP, respectively. The C concentrations of the tree layer leaves in middle-aged and mature forests were significantly higher than those of the roots, and the C concentrations of HAP at each stand age were significantly higher than those of HUP. This demonstrates that strategic nutrient allocation to leaves enhances ecosystem resilience and productivity, as leaves are the primary photosynthetic tissues in plants^26^. When nutrients are transferred from one tissue to another in the plant, the concentration of the elements can also be affected^27^, as shown by Zhang et al.^28^. Elements in plant tissues are affected by the dilution effect caused by increased leaf area, biomass, etc. In this study, the N concentrations of the tree layer leaves, and shrub layer leaves and HAP at each stand age were remarkably higher than those of other tree organs, and shrub organs and HUP, respectively. However, Yang et al.^29^ found that the C:N ratio showed the opposite trend, which was consistent with the results of research about the N concentrations of understory vegetation in *Pinus armandii* and *Larix principis-rupprechtii.* Furthermore, Zhang et al.^30^ obtained the same conclusion for the nutrient levels in 187 shrubs in southern China. This mainly relates to the functions of plant tissues; for example, the leaves of plants mainly perform carbon synthesis, while the stem and root are mainly mechanical support tissues for plants. Plants need to reasonably allocate limited nutrients to each tissue in a reasonable manner to maximize their growth capacity^31^. On the other hand, this means that the N elements in the vegetation layer of the entire natural *P. massoniana* forest ecosystem showed a high degree of synergy, all of which were the highest in plant leaves, and also affected the C:N ratio of the entire vegetation layer, making it the lowest in the leaves. This could be linked to the environmental conditions during its growth. Plants in drier places tend to allocate more N to their leaves to increase their photosynthetic rate and water use efficiency, nutrients are redistributed in response to stresses such as drought and nutrient deficiency^32^. We found that the soil C and N concentrations became lower with increased soil depth; the change was far more significant at the 0–10 cm soil depth than at other soil depths. It is consistent with Dafeng Hui's study on the vertical variation of soil carbon and nitrogen stoichiometry in tropical forests on Hainan Island, China^15^. This trend is largely due to the fact that most animals and microorganisms that enhance nutrient accumulation reside in the surface soil^30^. Meanwhile, affected by the nutrient return from the litter and the surrounding environmental factors, the soil nutrients first accumulate on the soil surface and are then diffused to deeper layers with water or other mesons. Therefore, the concentrations of C and N in the surface soil were higher than that of other soil depths. Changes in soil C and N affect plant nutrient uptake and utilization, which holds significant implications for forest management strategies.

Carbon (C) is the basis of plant growth, reproduction, and structure and accounting for about 50% of plant dry weight^33^. Nitrogen (N) is the major organ of all enzymes and chlorophyll in plants, and plays an important role in controlling carbon uptake and primary production^34^. The C:N ratio of plants is an important indicator of plant growth rate; usually, the higher the C:N ratio of plants, the slower their growth rate. This paper found that the C concentration and C:N ratios of the tree layer were significantly higher than those of the understory vegetation layer, but the N concentration presented an opposite trend. This indicates that there are differences in nutrient utilization among different plants, which have varying impacts on carbon sequestration and ecosystem productivity.The tree layer had a stronger photosynthesis than the understory vegetation layer, which resulted in accumulating more organic carbon, while the understory vegetation layer grew at a faster rate. Compared to woody plants, short-lived herbaceous plants require more nutrients for rapid growth^35,36^. The data presented in this study showed that the C concentration of litter was higher than that of the understory vegetation but smaller than that of the trees, and the N concentration was significantly higher than that of the understory vegetation layer and the tree layer. The reason for this is that the tree layer has the greatest capacity for carbon accumulation and the highest C concentrations. Therefore, the main reason why the C concentration of the tree layer was higher than that of other ecosystem layers is that the carbon accumulation capacity of the tree layer is the greatest. The tree and undergrowth vegetation layer are the primary sources of litter, and its decomposition is influenced by its composition. The decomposition process is usually slower when litter contains more lignin and cellulose. For example, litter in coniferous forests is more difficult to decompose than that in broadleaf forests^37,38^, so the C concentration of the litter is higher than that of the understory vegetation layer. Uncertainties in elemental budgets were much higher for N than C; for example, they may be influenced by exogenous N elements such as N deposition; to some extent, the N concentration may have been caused by the absolute accumulation of N in the litter decomposition process^39,40^. The N concentration of litter may be increased in some way by all of these. This further elucidates the complex interactions within forest ecosystems, offering insights for sustainable forest management practices that leverage natural nutrient cycling processes.

### Variation in C and N stoichiometry over the stand age sequence

The age of Forest stands had different influences on the C and N stoichiometry among various organs and layers of the ecosystem. In this study, we found that the N concentrations of tree branches and trunks showed a trend of rising then declining with stand age, and remarkable changes appeared in the young forest, in which the N concentrations were significantly lower than those in near-mature forests, but the C:N ratios of tree branches and trunks presented an opposite trend. The physiological metabolism and development of plants is greatly impacted by the absorption and utilization of nitrogen elements. To maintain high levels of amino acid and protein synthesis in plants, to promote plant growth, reduce the carbon–nitrogen ratio^8^, and delay plant aging, it is necessary to have sufficient nitrogen. This result demonstrated that the growth rate of branches and trunks in the tree layer of natural *P. massoniana* forest gradually accelerated from young forest to near-mature forest, reached its maximum value in the near-mature forest, and then slowly reduced the growth rate. Simultaneously, we also found that the C concentrations of all tissues of shrubs showed a first decline and then a increase with the stand age, and all showed that near-mature forests were significantly smaller than young forests. This means that the ability of all tissues of the shrub to accumulate carbon gradually weakens with age. The soil C:N ratio occupies an important position in the soil quality assessment index, and the higher the general C:N ratio, the slower the mineralization of soil organic matter^41^. The soil C:N ratio of the 0–10 cm soil gradually increased from 14.48 in young forests to 19.24 in mature forests was observed. On the one hand, this meant that the mineralization capacity of organic matter in topsoil gradually weakened with age. On the other hand, the topsoil C:N ratios in four age groups were greater than the average C:N ratios of global soils (13.33) and the average C:N ratios of Chinese soils (12.00–13.00)^42,43^. This indicated that the overall mineralization of organic matter in the topsoil was slow. This was affected by the composition and concentration of litter, especially the amount of lignin and cellulose in litter^44^.

It can be concluded that with increased stand age, the C concentration of the tree layer increased from young forest to near-mature forest and then decreased, and the C concentration of the understory vegetation layer and litter layer exhibited a decreased trend in the study. The combination of the growth rate of trees at different growth stages, the illumination conditions in the forest, and competition among trees may have caused this. Generally, the growth rate of trees is fast before maturity and then slows down. At the same time, the growth of the tree directly affects the illumination conditions in the forest, influencing the coverage, height, and species of understory vegetation, and the litter layer is comprehensively influenced by the tree layer and the understory vegetation layer^45^. The growth of the understory vegetation and the decomposition of litter gradually accelerates with age, which may be mainly related to forest humidity and species diversity. Humidity in the young forest stage is low, there are relatively few understory vegetation species, and litter mixing is not high. With increased stand age, species diversity improves gradually, which can provide better forest microclimate conditions that are beneficial to the growth of understory vegetation and litter decomposition. The N concentration at every layer of the entire ecosystem gradually increased with age was found. There was a high degree of synergy between the different layers. This could be linked to changes in nutrient dynamics and biodiversity^46,47^. The soil C and N concentrations and the C:N ratio gradually increased with the increasing of stand age^48^. Wang et al.^49^ studied the soil stoichiometric characteristics of *Larix principis-rupprechtii* plantations in Guandi Mountain and found that the total soil and all nitrogen were the largest in the near-mature forest, and the C:N ratios decreased with the increased stand age. Li et al.^50^ studied the variation of carbon and nitrogen stoichiometry in plants and soil following the stand age of *Robinia pseudoacaia* plantations and found that there was no obvious trend of soil C:N ratios with the increased stand age. It's possible that this is connected to tree species and the study area. Different study areas and tree species have different physical and chemical properties in the understory soil at different stand age^51,52^.

### Correlation of C and N stoichiometry among various ecosystem layers

The C and N concentrations and the C:N ratio across ecosystem layers were found to have significant or extremely significant correlations between and within each layer. In particular, the N showed a positive correlation at all layers of the ecosystem, among which there was a significant positive correlation between the tree layer and the soil layer, the understory vegetation layer and the litter layer, and the litter layer and the soil layer. The positive correlations across different ecosystem levels underscore the integrated nutrient cycling within the ecosystem, particularly nitrogen's crucial role in linking forest components from canopy to soil. This interconnection suggests a coordinated system that efficiently recycles nutrients to support forest growth and sustainability. Moreover, plants have developed adaptive strategies to optimize nutrient use amid environmental changes, emphasizing the dynamic balance between photosynthesis and mineral metabolism essential for maintaining ecosystem productivity and resilience against environmental fluctuations. Understanding these mechanisms is vital for enhancing ecosystem services and biodiversity conservation^53^.

The stoichiometric characteristics of the different layers of the ecosystem have some differences due to the different ecological functions performed. In this study, the C concentrations in understory vegetation layers were significantly positively correlated with the C concentrations in litter layers, but negatively correlated with the soil layers, and the C concentrations in litter layers were also significantly negatively correlated with the C concentrations in soil layers. It indicated that the nutrient cycle of the ecosystem was blocked or that the decomposition rate was slow in the process of nutrient decomposition and release from litters to the soil. This may be related to the composition of the litter. On the one hand, the composition of natural *P. massoniana* coniferous forest litter is given priority with the restraints of masson pine needles, in which the masson pine needles contain a lot of difficult- to-decompose components such as lignin, which affect the decomposition rate of litter. On the other hand, it may be caused by the comprehensive influence of multiple factors such as N deposition and climate change^54,55^. According to this study, the C:N ratio of the understory vegetation layer had a significant negative correlation with the C concentrations in the tree layer. This indicated that when the understory vegetation grew at a faster rate, the carbon accumulation of the tree layer increased, which was mainly due to competition among vegetation and was closely related to the carbon storage and carbon balance of the whole ecosystem^56,57^. In this study, there was a negative correlation between the C:N of the tree layer and the concentrations of C and N, and C:N ratio of the soil layer. According to the findings, the rate of soil mineralization and the release of nutrients decreased when the vegetation layer grew faster. This is mainly because plant growth needs to consume a lot of nutrients, and the source of nutrients for plants is in addition to their own photosynthesis; the other part is mainly obtained from the soil, so when the plant growth rate is relatively fast, the demand for soil nutrients increases, which will affect the accumulation of nutrients in the soil itself^58^. This exploration could shed light on strategies to enhance the efficiency of nutrient recycling within forest ecosystems, thereby contributing to their health and sustainability.

## Conclusions

To ascertain healthy and sustainable forest ecosystem functions and nutrient cycling, this study explored the C:N stoichiometry over time in different ecosystem layers of the natural *P. massoniana* forest in a subtropical region of China. The results showed that the concentration of C and N nutrients in each tissue of plants was highest in leaves. The nutrients found in the surface soil layer were significantly superior to those found in other deeper soil layers. The growth rate of branches and trunks in the tree layer gradually accelerated from young forest to near-mature forest, reached the maximum value in the near-mature forest, and then slowly reduced the growth rate. The mineralization capacity of organic matter in the surface soil layer gradually weakened with age and the overall mineralization of organic matter was slow. Overall, the tree layer had the greatest accumulation of organic carbon, while the understory vegetation showed a higher growth rate. The rate of litter decomposition and soil mineralization was relatively slow, and the nutrient cycling of the entire ecosystem was hindered to some extent. The C and N concentrations and the C:N ratio among ecosystem layers showed significant or extremely significant correlations. All layers of the ecosystem were positively correlated with N concentrations and there was high synergy throughout the natural *P. massoniana* forest ecosystem. These results provide a better understanding of the distribution patterns of stoichiometry in forest ecosystems linked with stand age and the theoretical basis for maintaining the sustainable development of forest ecosystems.

## Data Availability

The datasets generated during and/or analyzed during the current study are available from the corresponding author on reasonable request.
